# Skeletonization of radial and gastroepiploic conduits in coronary artery bypass surgery

**DOI:** 10.1186/1749-8090-2-26

**Published:** 2007-06-05

**Authors:** Rachel M Massey, Oliver J Warren, Michal Szczeklik, Sophie Wallace, Daniel R Leff, John Kokotsakis, Ara Darzi, Thanos Athanasiou

**Affiliations:** 1 Department of BioSurgery and Surgical Technology, Imperial College London, 10^th ^Floor QEQM Wing, St. Mary's Hospital, Praed Street, London, W2 1NY, UK

## Abstract

The use of a skeletonized internal thoracic artery in coronary artery bypass graft surgery has been shown to confer certain advantages over a traditional pedicled technique, particularly in certain patient groups. Recent reports indicate that radial and gastroepiploic arteries can also be harvested using a skeletonized technique. The aim of this study is to systematically review the available evidence regarding the use of skeletonized radial and gastroepiploic arteries within coronary artery bypass surgery, focusing specifically on it's effect on conduit length and flow, levels of endothelial damage, graft patency and clinical outcome. Four electronic databases were systematically searched for studies reporting the utilisation of the skeletonization technique within coronary revascularisation surgery in humans. Reference lists of all identified studies were checked for any missing publications. There appears to be some evidence that skeletonization may improve angiographic patency, when compared with pedicled vessels in the short to mid-term. We have found no suggestion of increased complication rates or increased operating time. Skeletonization may increase the length of the conduit, and the number of sequential graft sites, but no clear clinical benefits are apparent. Our study suggests that there is not enough high quality or consistent evidence to currently advocate the application of this technique to radial or gastroepiploic conduits ahead of a traditional pedicled technique.

## Background

In coronary artery bypass surgery (CABG), total arterial revascularisation may achieve superior long-term patency and improved survival rates, when compared to more traditional revascularisation techniques [1]. Clinical choice of arterial conduits should be based on patient characteristics, biological characteristics of the conduits, anatomical characteristics of coronary artery anastomotic targets and other technical factors (including harvesting technique and use of antispasmodic pharmacotherapy).

The predominant practice in cardiac surgery is to harvest the arterial conduit as a pedicled graft. Skeletonized conduits are arteries that have been dissected from all surrounding tissues, including accompanying veins, fascia, lymphatics and adipose tissue, leaving the adventitia as the outermost layer. The evidence for using a skeletonized internal thoracic artery (SKT-ITA) in coronary artery bypass graft surgery (CABG), as opposed to the less technically demanding pedicled conduit, has been recently examined [2]. Due to concerns over increased wound infection and sternal dehiscence rates, bilateral or unilateral left internal thoracic artery was traditionally avoided in obese patients and those with poorly controlled diabetes mellitus [3]. These views have been considered controversial, and arterial revascularization may be beneficial in these groups when skeletonization is performed [2,3].

Arterial revascularization often includes harvesting of radial or right gastroepiploic arteries. The radial artery (RA) was first used as an arterial conduit by Carpentier in 1973 [4]. However, initial angiographic studies revealed high occlusion rates at follow-up and the practice was largely abandoned until the revival of its use by Acar et al [5]. The gastroepiploic artery (GEA) was first used as an arterial conduit in cardiac surgery by Pym et al in 1984 [6]. Concerns have been raised regarding vessel spasm and luminal diameter in these arterial conduits. Both the RA and the GEA have more smooth muscles cells in the wall and are less elastic than the ITA [7] and the GEA has a higher tendency to spasm when compared to the ITA [8]. Furthermore, the different conduits respond to the same vasoconstrictors with varying magnitude. The RA responds more strongly in response to serotonin than does the ITA, and the GEA responds more vigorously than both other conduits to potassium, thromboxane A_2 _and Norepinephrine [9].

Initially both the radial and gastroepiploic arteries were harvested as pedicles. However recent reports indicate that radial [10-12] and gastroepiploic arteries [3,13,14] are also being harvested using a skeletonization technique. Advocates of RA and GEA skeletonization argue that it facilitates surgical manipulation, increases graft luminal diameter, reduces graft spasm and reduces the incidence of early graft stenosis (string sign).

### Anatomy, physiology and harvesting techniques for the skeletonized radial artery

The radial artery is the smaller and more direct of the two terminal branches of the brachial artery. Arising from the cubital fossa opposite the neck of the radius, it descends through the lateral aspect of the forearm, and enters the palm to anastomose with the deep branch of the ulnar artery to complete the deep palmar arch. The proximal RA lies in close proximity to the lateral cutaneous nerve of the forearm, and it's midpart lies close to the superficial branch of the radial nerve. Thus, both nerves are at risk during harvesting.

Taggart et al were the first to report RA skeletonization [15]. Skeletonization is technically demanding and can be performed using scissors and haemostasis clips or electrocautery/ultrasonic scalpel dissection. Some groups describe in-situ RA skeletonization [10], whilst others describe initial RA harvesting as a pedicle and then ex-vivo skeletonization using the ultrasonic scalpel [12].

### Anatomy, physiology and harvesting techniques for the skeletonized gastroepiploic artery

The right and left GEAs run between the layers of the greater omentum, supplying both surfaces of the stomach. Being a fourth order artery, the diastolic pressure and the flow rate in the GEA is lower than that of the internal thoracic artery. Furthermore, during periods of physiological stress the associated increase in sympathetic tone leads to blood flow being diverted away from the viscera to muscles and vital organs [16]. As a consequence if a GEA conduit is used in coronary revascularization, flow through the redirected GEA to the myocardium may be compromised [17]. As with other conduits, GEA skeletonization is more time consuming and technically demanding than pedicle harvest. Gagliardotto et al were the first to document skeletonization of the GEA [14]. Their initial technique using scissors and haemostasis clips has been recently modified, to utilise an ultrasonic scalpel [18-20].

### Aim and scope of article

The aim of this review article is to systematically examine the evidence regarding the use of skeletonized radial and gastroepiploic arterial conduits. We focus on the advantages and disadvantages of this technique in both vessels, with specific regard to conduit length, conduit flow, endothelial wall damage, graft patency and short and long term clinical outcomes.

## Materials and methods

### Literature search

A Medline, Ovid, Embase and Cochrane database search was performed to identify all studies concerned with the use of SKT-RAs or SKT-GEAs in cardiac surgery. The following MeSH headings were used: "radial artery", "gastroepiploic artery", "skeletonization", "skeletonized" and "cardiac", "surgery", "outcomes", "free flow" and "spasm". The 'related articles' function was utilised to broaden the search and all abstracts were scanned and reviewed. Based on the title and abstract of the publication, we retrieved articles containing clinical data on the use of skeletonized radial and gastroepiploic arteries in cardiac surgery. References of the articles acquired were also searched manually, to ensure no data was missed. The search was restricted to publications in the English Language. The latest date for this search was the 1^st ^January 2007.

### Data extraction and validation of the studies

Three reviewers (S.W., D.L. and M.S.) independently extracted the following data from each study; first author, year of publication, study population characteristics, study design, number of subjects, procedure type and the following outcomes of interest; conduit length or calibre, conduit flow, endothelial wall damage, early and late angiographic patency and morbidity and mortality associated with SKT-RA or SKT-GEA harvesting.

Articles were classified as case reports or series, and clinical studies. The clinical studies were further classified according to whether or not they were retrospective or prospective, in which case they must have a predefined outcome to be assessed, and whether or not they were comparative. We excluded studies which did not report on at least one of the outcome measures mentioned above, or which did not contain any clinical data. The studies were too heterogeneous to be combined for a formal meta-analysis, and therefore a systematic synthesis was undertaken.

## Results

### Study identification

**Figure 1:**
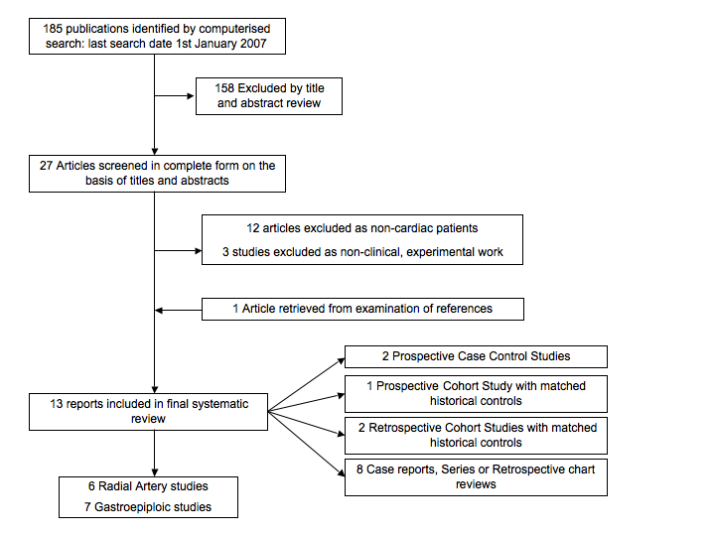
'Systematic search strategy'.

Figure 1 outlines the systematic search strategy and results. 27 articles and their references were investigated in full. This led to the exclusion of 14 studies, 11 non-cardiac papers and 3 due to a lack of clinical data. This left 13 articles, 6 focused on RA skeletonization [10-12,15,21,22] and 7 on GEA skeletonization [13,23,20,24]. There were no prospective, double blind, randomised controlled trials. However, there were 5 comparative studies: 2 prospective case control studies [13,21], 1 prospective cohort study with matched historical controls [12], and 2 retrospective cohort studies with matched historical controls [20,23]. These five studies are summarised in Table 1. The remaining 8 publications were either case reports, case series or retrospective cohort reviews [10,11,14,15,18,19,22,24]

**Table 1:** Comparative studies reporting skeletonization of the radial and gastroepiploic arteries

**Study**		**Conduit**	**Number of patients**		**Skeletonized Group**		**Key Findings**	**Adverse Events**
		
**Ref**	**Type**	**RA/GEA**	**Skel**	**Control**	**Age (Yrs ± S.D.)**	**Gender M/F**		
***Kamiya et al [13]***	A	GEA	70	98	60 ± 9.2	51/80	Significantly increases luminal diameter Composite grafts effective for multiple grafting	No deaths 15 Atrial Fibrillation
***Rukosujew et al [21]***	A	RA	20	20	n/a	N/a	Significantly increases harvesting time Length significantly increased by skeletonization with scissors Endothelial damage seen in all groups	Not recorded
***Amano et al [12]***	B	RA	131	112	65.8 ± 8.9	102/80	Proximal diameter of RA significantly larger in skeletonized conduits Significant increase in sequential RA grafting	2 MI, 2 Respiratory Failures, 4 CVA, 2 Mediastinitis, 2 Deaths
***Li et al [20]***	C	GEA	59	21	66.7 ± 8.8	46/19	No significant difference in harvest time or number of distal anastomoses	1 CVA, 1 Respiratory Failure, 1 Death
***Kamiya et al [23]***	C	GEA	168	60	65 ± 11.5	131/47	Functional patency significantly better in skeletonized group	20 Atrial Fibrillation, 1 MI, 2 Re-exploration for bleeding

Study and Study Type: A = Prospective Case Control Trial, B = Prospective Cohort Study with Case Matched Historical Control Trial, C = Retrospective Cohort Study with Case Matched Historical Control Trial

Conduit: RA = Radial Artery, GEA = Gastroepiploic Artery

Age: Yrs = Years, S.D. = Standard Deviation

Gender: M = Male, F = Female

Abbreviations; CVA = Cerebro-vascular Accident, MI = Myocardial Infarction, Skel = Skeletonized, Ref = Reference, n/a = Not available

### Case mix and patient demographics

The 13 articles reported on 1523 cardiac patients, of which 946 received either a skeletonized radial or gastroepiploic artery as an arterial conduit for coronary artery bypass grafting, and 577 were non-skeletonized controls. Within the treatment group 49% of patients received a skeletonized radial artery and 51% received a skeletonized gastroepiploic artery. Furthermore, patients were distributed relatively evenly between the types of bypass surgery they had; 32% had CABG performed on-pump, 59% off pump bypass surgery and in 9% this was not recorded. Pre-operative morbidity was recorded in 91% of patients receiving a skeletonized radial conduit, and of these 51% were recorded as diabetic. Pre-operative morbidity characteristics were only recorded in 18% of patients who underwent skeletonization of their gastroepiploic artery, but of these 62% were diabetic.

### How does skeletonization affect harvesting time?

Of the 946 patients who received a skeletonized conduit, only 16% had harvesting time and/or total operating time recorded as a comparative endpoint. All of this cohort received a radial artery conduit. Skeletonization is known to be more technically demanding, and thus it has been assumed that it increases the length of time for both conduit harvest and the procedure as a whole. Rukosujew et al compared skeletonization, using dissection with either scissors or ultrasonic scalpel, with a standard pedicled harvesting technique [21]. They demonstrated skeletonization takes significantly longer using either scissors (p < 0.001) or the ultrasonic scalpel (p < 0.01). However, they presented no data to see whether this translated into an increase in total operating time. Amano et al did not study harvesting time but noted no effect on total operating time for those patients undergoing skeletonization (p > 0.05) [12]. Unfortunately, none of the studies assessing GEA skeletonization comment on harvesting or total operating time. It would be interesting to compare the harvesting time required between SKT-RA and SKT-GEA as the anatomy suggests that GEA skeletonization would be more time consuming than radial skeletonization.

### Does skeletonization influence sequential grafting or mean number of distal anastomotic sites?

Many authors believe that additional length provided by skeletonization improves the ease of grafting the RA, facilitates sequential grafting and increases the number of possible distal anastomoses. Amano et al report that sequential RA grafting was undertaken in 33.6% of the skeletonized group compared with just 17% in the pedicled group (p < 0.05) [12]. Rukosujew et al comment that skeletonization of the vessel made identification of the optimal anastomotic site easier and therefore made sequential grafting easier but did not gather any quantitative data or perform any statistical analysis [21], a point supported by Hirose et al [10]. Whether skeletonization of the GEA influences sequential grafting or mean number of distal anastomotic sites has yet to be assessed.

### Does skeletonization have an effect on vessel endothelium?

Critics of the skeletonization technique argue that the method is more likely to lead to vessel damage both macroscopically and microscopically and that microscopic endothelial damage may predispose to early graft stenosis. Whilst we appreciate the possible role of publication bias, none of the studies in our review reported macroscopic RA or GEA dissection or injury when using the skeletonization technique.

The highest quality study assessing vessel endothelial damage was performed by Rukosujew et al [21]. They used scanning electron microscopy to look for possible endothelial damage in conduit surplus to the procedure. Minor endothelial damage was observed in all vessels, regardless of harvesting technique and was therefore considered to be not clinically significant. With respect to severe endothelial damage, skeletonization was associated with significantly higher levels of damage than the pedicled technique (p < 0.01). Furthermore, the most severe endothelial damage was found in vessels skeletonized with the harmonic scalpel as opposed to with scissors. They concluded that skeletonization with harmonic scalpel did not result in additional length and was associated with more endothelial damage than scissors. Other groups have studied endothelial damage in conduits skeletonized with a harmonic scalpel. Fukata et al assessed its effect on the vessel wall by looking at the maximum depth of thermal degeneration [25]. The study demonstrated that thermal degeneration was limited to the vessel's connective tissue and did not affect the media or intima in vessels used in a clinical setting.

### Does skeletonization influence angiographic patency?

#### Radial artery

Historical questions regarding early angiographic patency of the RA prevented its use in coronary artery bypass surgery for 20 years and studies still put early RA graft stenosis rates at 5–7% [26,27]. Patency data is available for 39% of the patients undergoing radial artery skeletonization. Despite the data being from relatively heterogeneous sources, and recorded over a range of 3 to 12 months post surgery, the patency rates of 95.2–98.6% are relatively consistent between the different studies. Amano et al have reported the largest single cohort of these patients [12]. Angiography was performed within 3 months of surgery in 96 treated patients and 76 controls. The quality of anastomosis was graded according to Fitzgibbon's classification [28]. Although there was no significant difference in overall graft patency rates between groups, rates of moderate stenosis were statistically higher in the pedicle group (p < 0.0014). Hirose et al also examined early angiographic patency in patients with SKT-RA conduits, comparing them to other non-skeletonized conduits at 3 months follow-up [10]. They found early perfect patency rates to be 96%, compared to 95.1% for Left ITA, 93.8% for Right ITA and 93.1% for GEA. A year later the same group performed an observational study in which angiography was carried out at one year [22]. They found graft patency rates of 95.2% in the SKT-RA, which compared favourably to data on other arterial conduits.

#### Gastroepiploic artery

Patency data was presented for 43% of the patients undergoing GEA skeletonization. This data is recorded over a range of 2 weeks to 12 months post surgery and gives patency rates ranging from 97.2–98.1%. Kamiya et al report the largest cohort of SK-GEA patients (n = 105) in whom follow-up angiography was performed [23]. They assessed patency in both SKT-GEA and pedicled vessels at 2 weeks post-operatively and demonstrated a statistically significant improvement in functional patency in those who had undergone skeletonization of their GEA, compared to controls (p < 0.02). There was also less flow competition and less graft stenosis. Ryu et al in an observational, non-comparative study report an immediate patency of SKT-GEA (used as a composite graft with Left ITA) of 97% [3], a figure supported by work performed elsewhere [19].

### Does skeletonization affect conduit length and calibre?

One of the most frequently cited justifications for skeletonization is that it can provide additional conduit length, and several groups report that the vessel appears longer on visual inspection, but fail to support this with any statistical analysis [10-12].

Rukosujew et al found that skeletonization of the radial artery yielded a significantly longer graft (20.8 ± 1.5 cm versus 19.1 ± 0.9 cm, p < 0.01) but only if performed using scissors and clips, not if performed with the ultrasonic scalpel [21]. Furthermore, whilst the difference in length was statistically significant, one could question the clinical significance of a 1 cm difference in conduit length. Unfortunately, they present no data on how this difference may have impacted on the procedure itself. Amano et al measured conduit diameter as opposed to length and found the RA was significantly larger in the skeletonized group than in the pedicle group (3.3 ± 0.3 mm vs. 3.1 ± 0.3 mm, p < 0.001) [12]. This finding has been replicated elsewhere, even when skeletonization was performed ex-vivo [29].

There is no quantitative data comparing SKT-GEA length or diameter with a pedicle GEA. Several authors however report increased vessel size on visual inspection but present no quantitative data to support this [18,20,23,24].

### Does skeletonization have any affect on morbidity or mortality?

The cumulative data on adverse events in all patients are recorded in Table 2. The complication rates for CABG using standard harvesting techniques, and CABG using either skeletonized RA or GEA conduits seem to be comparable [30].

**Table 2:** Complication rates in patients undergoing surgery with skeletonized conduits

**Complications**	**RA** **[n = 464 (%)]**	**GEA** **[n = 482 (%)]**	**Total** **[n = 946 (%)]**
***Not Recorded***	424 (91)	438 (91)	862 (91)
***Post operative MI***	7 (1.5)	1 (0.21)	8 (0.85)
***Arrhythmia***	2 (0.4)	35 (8)	37 (3.9)
***CVA***	13 (2.8)	1 (0.2)	14 (1.7)
***Re-exploration for bleeding***	3 (0.6)	1 (0.2)	4 (0.42)
***Mediastinitis***	6 (1.2)	0	6 (0.63)
***In hospital death***	3 (0.6)	1 (0.2)	4 (0.42)
***Respiratory failure***	10 (2.1)	1 (0.1)	11 (1.1)

Conduit: RA = Radial Artery, GEA = Gastroepiploic Artery, N = Number

Complications: CVA = Cerebro-vascular Accident, MI = Myocardial Infarction

Amano et al compared post-operative outcomes in patients treated with skeletonized RA conduits, versus those with pedicled conduits [12]. They found no significant difference in rates of post-operative myocardial infarction, respiratory failure, stroke, re-exploration for bleeding, mediastinitis or in-hospital deaths. Other studies record post-operative outcomes, but without any comparison with controls [11,22].

GEA harvesting involves opening the peritoneum, and as such one could postulate that it has a higher overall risk than radial artery or saphenous vein harvest. By opening the abdominal cavity the patient is at risk of complications such as early small bowel obstruction [31] and incisional hernia [32]. Furthermore, by skeletonizing the GEA, one may increase the amount of time the abdomen is open, thus increasing morbidity and mortality rates. To address these issues, Li et al compared the risk of major complications postoperatively between treated and control groups and showed no significant difference in the rates of low output syndrome, post-operative myocardial infarction, arrhythmia, stroke, re-exploration and mediastinitis [20]. Kamiya et al followed all surviving patients and demonstrated no delayed abdominal complications such as ileus, bowel obstruction, gastric perforation or incisional hernia [13]. In addition there was no significant difference in operation time, intubation time, or length of intensive care unit or hospital stay. Regrettably, we found no further studies that looked specifically at duration of intensive care or hospital stay.

### Does skeletonization facilitate novel usage of the vessel?

In recent years there has been increased interest in minimally invasive surgical techniques to reduce peri-operative morbidity and mortality rates [33]. One such development has been the use of automatic anastomotic devices. Currently, these devices are limited to the deployment of saphenous vein grafts, and cannot be used for arterial conduits as these are usually harvested as a pedicle. Watanabe et al harvested the RA in a completely skeletonized fashion to facilitate usage of the St. Jude Medical Symmetry Aortic Connector System for automatic anastomosis [11]. In their 10 cases, they achieved satisfactory anastomoses without any complication or technical difficulty in all cases and reported no cardiac related event in a 10.3 +/- 2.9 month follow up. The fact that skeletonization may facilitate the use of an anastomotic device is interesting, and there may be additional benefits to using skeletonized vessels that are yet to be fully established.

### Does skeletonization of the GEA influence flow competition with the native coronary artery?

As discussed earlier, there have been concerns regarding the use of the GEA due to a potential for flow competition with the native coronary artery. Previous studies have shown that diastolic pressure is significantly lower in the GEA than the ITA, making it more prone to insufficient flow if there is competitive coronary flow [34]. There is also evidence that there is a relationship between GEA diameter and graft patency, with smaller GEA diameter related to low GEA flow. Ochi et al suggest that the GEA luminal diameter should be 2–3 mm at the anastomotic point to generate adequate perfusion pressures to avoid competitive flow [35]. Whilst most authors conclude that skeletonization reduces competitive flow, this assertion can only be objectively assessed in vivo by postoperative angiography. In the reported series of Ryu et al, 2 out of 37 patients with SKT-GEA grafts to the right coronary artery with moderate proximal stenosis (≤ 70%) developed the "string-sign" suggestive of graft spasm [3]. Kamiya et al's group demonstrated flow competition or diffuse string sign of the GEA graft in 2 of 105 patients (2%) in the SKT-GEA group and 6 of 40 patients (15%) in the pedicled GEA group [23].

### Comment

We have synthesised all of the available data within the cardiac literature on skeletonization of the two key non-thoracic arterial conduits and have attempted to elucidate any clear advantages. Ever since Keeley first reported skeletonization of the internal thoracic artery in 1987 [36] and evidence appeared suggesting that it is beneficial in patients with diabetes mellitus [2] there has been interest in the potential benefits of skeletonization of other conduits.

The main arguments for skeletonization of both the RA and GEA are that it increases vessel length, improves early angiographic patency rates and improves vessel flow and with respect to the GEA reduces competitive flow with the native coronary artery. Critics argue that the technique increases the likelihood of vessel damage, both macroscopic and microscopic, makes vessel spasm more likely and as the harvesting technique takes longer, it lengthens the overall procedure length with a possible subsequent effect on patient morbidity and mortality. Most of these inferences are based on data from non-randomised observational studies, which limit their wider applicability to clinical practice.

Whilst skeletonization may increase the time taken to harvest the conduit we see no evidence that skeletonization, in-situ or ex-vivo, increases the total operating time. This is not unexpected as conduit harvest is often carried out concurrent to access and preparation of the operative field. Certainly, it appears from our data synthesis that skeletonized conduits can be employed in both on- and off-pump bypass surgery.

Whilst one study did show a statistically significant increase in RA conduit length, this study was underpowered, non-randomised and the difference in length was 1 cm [21]. It is difficult to extrapolate this additional length to any clinical advantage. Furthermore, no studies take account of patient height, weight or body surface area all of which are likely to impact upon vessel length and calibre.

Vessel damage as a result of skeletonization is a frequently cited reason for not employing this technique to harvest vessels. However, the amount and quality of data we have been able to extract pertaining to harvesting injury is disappointing. None of the studies included in our review report on how frequently skeletonization of the conduit led to it being discarded prior to grafting. Studies of the ITA that show higher rates of endothelial damage associated with skeletonization are not necessarily associated with poorer graft patency or function [37-39]. Whilst one of the studies in our review did show evidence of endothelial injury after skeletonization, this was not correlated with any clinical outcomes [29]. Recently, Matsumoto et al performed an experimental study assessing the effect of skeletonization with an ultrasonic scalpel on both the ITA and GEA. Although this study has not been included in our data synthesis, as it contains no clinical data, we felt it contained some important results. They provide the first evidence that ultrasonic skeletonization is probably more traumatic to endothelial function than a conventional harvesting technique [40], and concluded that those who perform skeletonization should only employ vasodilators whose mode of action is not reliant on intact endothelial function to improve graft patency in the early post operative period.

Our study suggests that skeletonization of both RA and GEA does not have an adverse effect on angiographic patency at one year follow-up; in fact some report it may improve it [22,23]. This may seem counterintuitive, as increased surgical manipulation is associated with reduced patency. Moderate stenosis in the pedicle group was found to occur more commonly by Amano et al [12] but it is not clear whether this occurred within the graft or at the site of anastomosis, which obviously has totally different implications. These results should be treated with caution, as they originate from centres with experience and expertise in what is a technically demanding procedure, however, some are convinced of a potential benefit and now only perform RA harvest in a skeletonized manner [12].

Our data synthesis shows no apparent effect of GEA or RA skeletonization on morbidity or mortality rates overall. None showed discrepancies between in-hospital deaths, postoperative myocardial infarction, stroke, or renal failure and there is no data to support the supposition that increased harvest time lead to increased complication rates. However there do appear to be differences in the rates of arrhythmia, CVA and respiratory failure between the two groups. The total number of patients in whom specific complications are recorded is small which may explain this. With specific regard to arrhythmia in some studies atrial fibrillation is included as an arrhythmia whilst in others only 'serious arrhythmia' is recorded.

The majority of GEA usage, skeletonized or not, occurs in Japan. There are very few studies objectively assessing parameters such as increased calibre and improved vessel flow as a result of skeletonization although authors do invariably comment on observed improvements. The consensus amongst skeletonization advocates is that measuring vessel calibre, length or diameter is not practical and increases are demonstrated functionally by increasing the amount of sequential grafting and the ability to graft to territories such as the circumflex and left anterior descending arteries, which is not normally accessible with the pedicled GEA. However, these are quantitative outcomes, and should be measured objectively and compared to pedicled controls before this technique can be judged to be superior to the traditional pedicled technique.

Lastly, previous review of skeletonization of the ITA suggested reduced post-operative infection rates particularly in diabetic patients [2]. 51% of patients undergoing RA skeletonization and 62% of patients undergoing GEA skeletonization were reported as diabetic. Although no authors report if there is specific advantage to skeletonization in these patients it may be that skeletonization is favoured as a technique in diabetics on the basis of previous research, and thus they are now over-represented within the literature. We would advise caution on the presumptive transferability of the findings of the ITA studies, and suggest that the data regarding the best harvesting technique in diabetic patients undergoing RA or GEA harvesting is inconclusive.

### Limitations and recommendations for further research

The major limitation of our systematic review is that the current evidence is relatively poor; whilst the number of patients within the literature is relatively high, they are scattered between 13 different reports, many of which are retrospective and non-comparative. Due to a paucity of prospective, comparative studies, very few definitive benefits or risks of skeletonization can be reported. We are unable to perform meta-analysis due to profound heterogeneity within the literature. However, the primary value of our manuscript is to give interim recommendations to those currently practicing, highlight the deficiencies within the literature and to guide future research. To this end we have formulated recommendations using the 'EPICOT' guidelines [41]. Firstly, having reviewed all the data, it is clear that future research is required. Whilst a double blind randomised control trial is impossible, due to surgeons being aware of which technique they are employing, a trial where patients and data collectors were blinded to the method of harvest and patients were randomised to either a skeletonized or pedicled group could be performed. Outcomes must include histological appearance, and short-, mid- and long-term clinical and angiographic patency outcomes. Research should focus on patient populations that may benefit from this technique most. We feel this may be diabetic patients with multivessel coronary disease, who require total arterial revascularization, but in whom bilateral ITAs should not be harvested.

## Conclusion

Skeletonization of the radial and gastroepiploic arteries is a relatively new approach. There appears to be some evidence that skeletonization may improve angiographic patency, when compared with pedicled vessels in the short to mid-term. We have found no suggestion of increased complication rates or increased operating time. Skeletonization may increase the length of the conduit, and the number of sequential graft sites, but no clear clinical benefits are apparent. We cannot currently advocate the application of this technique to radial or gastroepiploic conduits ahead of a traditional pedicled technique.

## Competing interests

The author(s) declare that they have no competing interests.

## Authors' contributions

RM, OW and SW were responsible for the study design, data interpretation, manuscript drafting and for important intellectual content. SW, DL, MS and JK were responsible for the collection, extraction and synthesis of data. AD was responsible for providing important intellectual content throughout the manuscript's production and for approval of the final version. TA is the guarantor. His involvement was critical to every phase of this work and he had access to the data and controlled the decision to publish. All authors read and approved the final manuscript.

## Funding

This study was undertaken as part of ongoing research at the Department of BioSurgery and Surgical Technology, Imperial College London, and did not receive separate funding.
